# Crystal structure of poly[di­chlorido­(μ-2,5-di­carb­oxy­benzene-1,4-di­carboxyl­ato-κ^2^ *O* ^1^:*O* ^4^)bis­[μ-4′-(pyridin-3-yl)-4,2′:6′,4′′-terpyridine-κ^2^ *N* ^1^:*N* ^4′^]dizinc]

**DOI:** 10.1107/S2056989016016285

**Published:** 2016-10-28

**Authors:** Yue Tian, Sha-Sha Xu, Jian Su, Yang Zhang, Shao-Shuai Zhao, Yu-Peng Tian

**Affiliations:** a Department of Chemistry, Key Laboratory of Functional Inorganic Materials Chemistry of Anhui Province, Anhui University, Hefei 230601, People’s Republic of China

**Keywords:** crystal structure, 4′-(pyridin-3-yl)-4,2′:6′,4′′-terpyridine, zinc(II) complex, coord­ination polymer

## Abstract

In the title polymeric Zn^II^ complex, [Zn_2_(C_10_H_4_O_8_)Cl_2_(C_20_H_14_N_4_)_2_]_*n*_, the Zn^II^ cations are bridged by both 2,5-di­carb­oxy­benzene-1,4-di­carboxyl­ate dianions and 4′-(pyridin-3-yl)-4,2′:6′,4′′-terpyridine ligands, forming ladder-like polymeric chains propagating along [1-10]. The Cl^−^ anion further coordinates the Zn^II^ cation to complete a distorted tetra­hedral environment. In the 4′-(pyridin-3-yl)-4,2′:6′,4′′-terpyridine ligand, the three sideward pyridine rings are twisted with respect to the central pyridine ring by 39.27 (12), 14.89 (13) and 3.36 (13)°, respectively. In the crystal, classical O—H⋯N hydrogen bonds and weak C—H⋯O and C—H⋯Cl hydrogen bonds link the chains into a three-dimensional supra­molecular architecture. π–π stacking is observed between the pyridine and benzene rings of neighbouring polymeric chains, with a centroid-to-centroid distance of 3.7280 (14) Å.

## Abstract 2

Both the 2,5-di­carb­oxy­benzene-1,4-di­carboxyl­ate dianions and pyridyl-terpyridine ligands bridge the Zn^II^ atoms, forming a ladder-like polymeric complex.

## Chemical context   

Coordination polymers (CPs) represent a class of crystalline materials which consist of different ligands inter­connected by metallic nodes (Yaghi & Li; 1995 ▸; Hinter­holzinger *et al.*, 2012 ▸). Compared to traditional inorganic materials, CPs have fascinating structures with regular pore shape and size obtained by rational design (Kepert, 2006 ▸; Brammer, 2004 ▸). In addition, studies over several decades have revealed that CPs have multi-functional applications such as gas storage and separation (Rosi *et al.*, 2003 ▸; Jiang *et al.*, 2013 ▸), chemical purification (Li *et al.*, 2012 ▸), catalysis (Seo *et al.*, 2000 ▸), and sensors (Kreno *et al.*, 2012 ▸), etc.
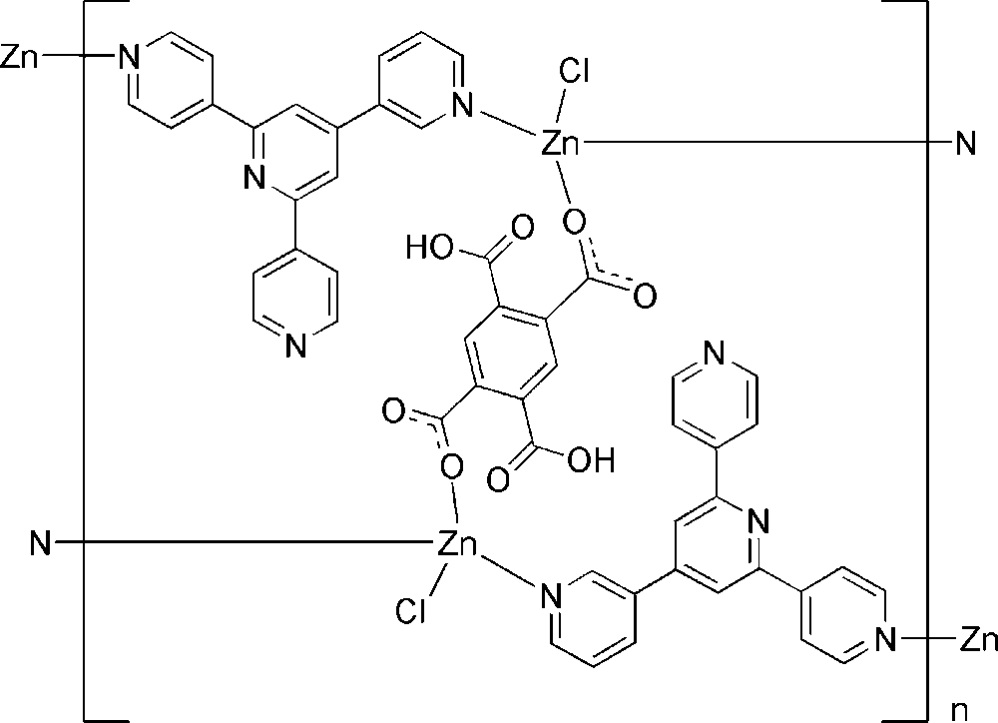


Pyridine-containing compounds, such as 4,2′:6′,4′′-terpyridine derivatives, are of great importance in the design of organic ligands, because conjugated polypyridyl ligands can form a better rigid plane and improve the stability of the network (Hancock, 2013 ▸; Li *et al.*, 2011 ▸; Bhaumik *et al.*, 2011 ▸). As a rigid planar and triangular ligand, 4′-(3-pyrid­yl)-4,2′:6′,4′′-terpyridine (344-pytpy; Housecroft, 2014 ▸) is different from commonly employed polypyridyl ligands such as 1,3,5-tri(4-pyrid­yl)-2,4,6-triazine (Ma & Coppens, 2003 ▸; Kumazawa *et al.*, 2003 ▸), which have been widely studied in the field of coordination chemistry. Its rigidity and trigonal geometry may lead to the formation of nanosized cages and porous frameworks enclosing cavities and channels (Li *et al.*, 2008 ▸; Yoshizawa *et al.*, 2004 ▸; Dai *et al.*, 2008 ▸). 1,2,4,5-Benzene­tetracarbonic acid (H_4_bta) is frequently employed due to the rich coordination binding sites of the carboxyl­ate groups (Hou *et al.*, 2011 ▸). We selected 344-pytpy and 1,2,4,5-benzene­tetra­carbonic acid as the organic linkers which, when assembled with Zn cations, resulted in the title coordination polymer [Zn_2_(344-pytpy)_2_(H_2_bta)Cl_2_]_*n*_.

## Structural commentary   

**Figure 1:**
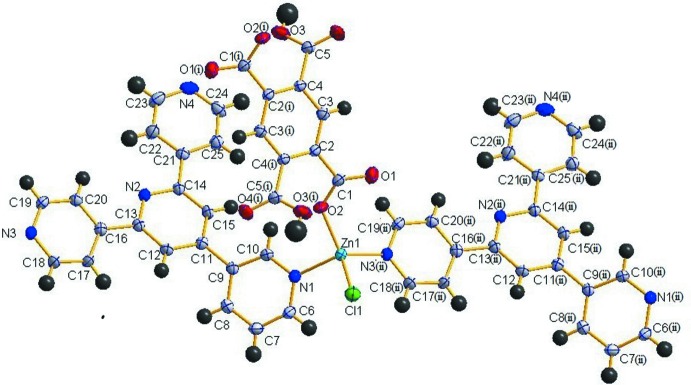
Part of the polymeric structure of the title complex [symmetry codes: (i) −*x*, −*y* + 1, −*z* + 2; (ii) *x* + 1, *y* − 1, *z*]. Displacement ellipsoids are drawn at the 50% probability level.

**Figure 2:**
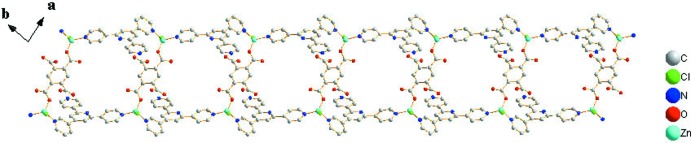
Part of the polymeric chain.

As shown in Fig. 1 ▸, the asymmetric unit of the title compound contains one Zn^II^ cation, one 344-pytpy ligand, a half of an H_2_bta^2−^ anion and one coordinating Cl^−^ anion. The Zn^II^ atom is four-coordinated by two nitro­gen atoms (N1, N3) from two different 344-pytpy ligands [Zn1—N1 = 2.070 (2) and Zn1—N3 = 2.0217 (18) Å], one oxygen atom (O2) from an H_2_bta^2−^ anion [Zn1—O2 = 1.9171 (16) Å] and one Cl atom [Zn1—Cl1 = 2.2278 (7) Å] in a distorted tetra­hedral coordination geometry. The bond lengths around Zn1 are similar to those reported by Wang *et al.* (2009 ▸). The *X*—Zn—*X* (*X* = N, O or Cl atom) angles range from 97.21 (8) to 115.73 (8)° and the tetra­hedron edge lengths range from 2.992 (3) to 3.591 (2) Å. Each 344-pytpy ligand act as 2-connecting node, linking two Zn atoms by the outer N-terminal atoms (N1, N3), the central and another outer pyridine N atom (N2, N4) are free. The H_2_bta^2−^ anion is located on an inversion center, and bridges two Zn^II^ atoms through the two carboxyl­ate groups. In this way, chains propagating along [1
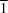
0] are formed (Fig. 2 ▸).

## Supra­molecular features   

In the crystal, classical O—H⋯N hydrogen bonds, weak C—H⋯O and C—H⋯Cl hydrogen bonds (Table 1 ▸) link the chains into a three-dimensional supra­molecular architecture. π–π stacking is observed between the N3-pyridine ring and benzene ring of the neighboring chain, with a centroid-to-centroid distance of 3.7280 (14) Å.

### Synthesis and crystallization   

4′-(3-Pyrid­yl)-4,2′:6′,4′′-terpyridine was synthesized accord­ing to a literature method (Yang *et al.*, 2014 ▸). 344-pytpy (0.0310 g, 0.1 mmol), ZnCl_2_ (0.0136 g, 0.1 mmol) and 1,2,4,5-benzene­tetra­carbonic acid (0.0254 g, 0.1 mmol) were adequately dispersed in 10 mL of distilled water, and then the mixture was sealed and heated to 453 K for three days under hydro­thermal conditions. The vial was then allowed to cool to room temperature. Colorless block-shaped crystals were collected (0.010 g, yield 38.9%, based on Zn).

### Refinement   

Crystal data, data collection and structure refinement details are summarized in Table 2 ▸. All H atoms were placed in geometrically idealized positions and treated as riding, with C—H = 0.93 and O—H = 0.82 Å, and with *U*_iso_(H) = 1.2*U*_eq_(C) and 1.5*U*_eq_(O).

## Supplementary Material

Crystal structure: contains datablock(s) global, I. DOI: 10.1107/S2056989016016285/xu5893sup1.cif

Structure factors: contains datablock(s) I. DOI: 10.1107/S2056989016016285/xu5893Isup2.hkl

Supporting information file. DOI: 10.1107/S2056989016016285/xu5893Isup3.cdx

CCDC reference: 1509671

Additional supporting information: crystallographic information; 3D view; checkCIF report

## Figures and Tables

**Table 1:** Hydrogen-bond geometry (Å, °)

*D*—H⋯*A*	*D*—H	H⋯*A*	*D*⋯*A*	*D*—H⋯*A*
O3—H3*A*⋯N4^ii^	0.82	1.83	2.633 (3)	167
C15—H15⋯O1^iii^	0.93	2.46	3.383 (3)	172
C17—H17⋯Cl1^iv^	0.93	2.75	3.678 (3)	173
C22—H22⋯O4^v^	0.93	2.59	3.305 (4)	134
C25—H25⋯O1^iii^	0.93	2.34	3.267 (3)	175

Symmetry codes: (ii) 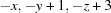; (iii) 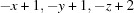; (iv) 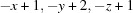; (v) 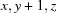.

**Table 2:** Experimental details

Crystal data	
Chemical formula	[Zn_2_(C_10_H_4_O_8_)Cl_2_(C_20_H_14_N_4_)_2_]
*M*_r_	537.24
Crystal system, space group	Triclinic, *P* 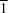
Temperature (K)	296
*a*, *b*, *c* (Å)	8.6557 (6), 12.1432 (8), 12.5842 (9)
α, β, γ (°)	61.396 (1), 74.216 (1), 75.411 (1)
*V* (Å^3^)	1105.83 (13)
*Z*	2
Radiation type	Mo *K*α
μ (mm^−1^)	1.27
Crystal size (mm)	0.23 × 0.22 × 0.17

Data collection	
Diffractometer	Bruker SMART APEX CCD
Absorption correction	Multi-scan (*SADABS*; Bruker, 2000 ▸)
*T*_min_, *T*_max_	0.758, 0.813
No. of measured, independent and observed [*I* > 2σ(*I*)] reflections	7896, 3840, 3435
*R*_int_	0.018
(sin θ/λ)_max_ (Å^−1^)	0.595

Refinement	
*R*[*F* ^2^ > 2σ(*F* ^2^)], *wR*(*F* ^2^), *S*	0.029, 0.080, 1.07
No. of reflections	3840
No. of parameters	317
H-atom treatment	H-atom parameters constrained
Δρ_max_, Δρ_min_ (e Å^−3^)	0.32, −0.27

Computer programs: *SMART* and *SAINT* (Bruker, 2000 ▸), *SHELXS97* and *SHELXTL* (Sheldrick, 2008 ▸) and *SHELXL2013* (Sheldrick, 2015 ▸).

## supplementary crystallographic information

### Crystal data

**Table:**

[Zn_2_(C_10_H_4_O_8_)Cl_2_(C_20_H_14_N_4_)_2_]	*Z* = 2
*M**_r_* = 537.24	*F*(000) = 546
Triclinic, *P*1	*D*_x_ = 1.613 Mg m^−^^3^
Hall symbol: -P 1	Mo *K*α radiation, λ = 0.71073 Å
*a* = 8.6557 (6) Å	Cell parameters from 4113 reflections
*b* = 12.1432 (8) Å	θ = 2.5–26.8°
*c* = 12.5842 (9) Å	µ = 1.27 mm^−^^1^
α = 61.396 (1)°	*T* = 296 K
β = 74.216 (1)°	Block, colorless
γ = 75.411 (1)°	0.23 × 0.22 × 0.17 mm
*V* = 1105.83 (13) Å^3^	

### Data collection

**Table:**

Bruker SMART APEX CCD diffractometer	3840 independent reflections
Radiation source: fine-focus sealed tube	3435 reflections with *I* > 2σ(*I*)
Graphite monochromator	*R*_int_ = 0.018
phi and ω scans	θ_max_ = 25.0°, θ_min_ = 1.9°
Absorption correction: multi-scan (SADABS; Bruker, 2000)	*h* = −10→10
*T*_min_ = 0.758, *T*_max_ = 0.813	*k* = −14→12
7896 measured reflections	*l* = −14→14

### Refinement

**Table:**

Refinement on *F*^2^	Primary atom site location: structure-invariant direct methods
Least-squares matrix: full	Secondary atom site location: difference Fourier map
*R*[*F*^2^ > 2σ(*F*^2^)] = 0.029	Hydrogen site location: inferred from neighbouring sites
*wR*(*F*^2^) = 0.080	H-atom parameters constrained
*S* = 1.07	*w* = 1/[σ^2^(*F*_o_^2^) + (0.043*P*)^2^ + 0.4805*P*] where *P* = (*F*_o_^2^ + 2*F*_c_^2^)/3
3840 reflections	(Δ/σ)_max_ = 0.001
317 parameters	Δρ_max_ = 0.32 e Å^−^^3^
0 restraints	Δρ_min_ = −0.27 e Å^−^^3^

### Special details

**Table:**

Geometry. All esds (except the esd in the dihedral angle between two l.s. planes) are estimated using the full covariance matrix. The cell esds are taken into account individually in the estimation of esds in distances, angles and torsion angles; correlations between esds in cell parameters are only used when they are defined by crystal symmetry. An approximate (isotropic) treatment of cell esds is used for estimating esds involving l.s. planes.
Refinement. Refinement of F^2^ against ALL reflections. The weighted R-factor wR and goodness of fit S are based on F^2^, conventional R-factors R are based on F, with F set to zero for negative F^2^. The threshold expression of F^2^ > 2sigma(F^2^) is used only for calculating R-factors(gt) etc. and is not relevant to the choice of reflections for refinement. R-factors based on F^2^ are statistically about twice as large as those based on F, and R- factors based on ALL data will be even larger.

### Fractional atomic coordinates and isotropic or equivalent isotropic displacement parameters (Å^2^)

**Table:**

	*x*	*y*	*z*	*U*_iso_*/*U*_eq_	
C1	0.3397 (3)	0.4695 (2)	0.8900 (2)	0.0279 (5)	
C2	0.1613 (3)	0.4839 (2)	0.9422 (2)	0.0218 (5)	
C3	0.1062 (3)	0.3951 (2)	1.0600 (2)	0.0239 (5)	
H3	0.1774	0.3240	1.1002	0.029*	
C4	−0.0533 (3)	0.4105 (2)	1.11919 (19)	0.0219 (5)	
C5	−0.0971 (3)	0.3173 (2)	1.2513 (2)	0.0306 (6)	
C6	0.6841 (3)	0.8624 (2)	0.5920 (2)	0.0297 (5)	
H6	0.7535	0.8319	0.5380	0.036*	
C7	0.6660 (3)	0.9894 (2)	0.5599 (2)	0.0317 (6)	
H7	0.7220	1.0438	0.4860	0.038*	
C8	0.5628 (3)	1.0346 (2)	0.6398 (2)	0.0280 (5)	
H8	0.5457	1.1208	0.6186	0.034*	
C9	0.4845 (3)	0.9514 (2)	0.7519 (2)	0.0228 (5)	
C10	0.5101 (3)	0.8245 (2)	0.7761 (2)	0.0269 (5)	
H10	0.4580	0.7676	0.8505	0.032*	
C11	0.3721 (3)	0.9962 (2)	0.8406 (2)	0.0234 (5)	
C12	0.2691 (3)	1.1105 (2)	0.7983 (2)	0.0259 (5)	
H12	0.2725	1.1608	0.7143	0.031*	
C13	0.1608 (3)	1.1490 (2)	0.8828 (2)	0.0236 (5)	
C14	0.2517 (3)	0.9694 (2)	1.0459 (2)	0.0230 (5)	
C15	0.3640 (3)	0.9256 (2)	0.9672 (2)	0.0254 (5)	
H15	0.4331	0.8497	0.9987	0.030*	
C16	0.0390 (3)	1.2655 (2)	0.8443 (2)	0.0237 (5)	
C17	0.0453 (3)	1.3573 (2)	0.7232 (2)	0.0303 (6)	
H17	0.1331	1.3513	0.6632	0.036*	
C18	−0.0777 (3)	1.4565 (2)	0.6922 (2)	0.0299 (6)	
H18	−0.0715	1.5165	0.6104	0.036*	
C19	−0.2098 (3)	1.3857 (2)	0.8926 (2)	0.0251 (5)	
H19	−0.2965	1.3958	0.9514	0.030*	
C20	−0.0910 (3)	1.2841 (2)	0.9303 (2)	0.0254 (5)	
H20	−0.0971	1.2276	1.0133	0.030*	
C21	0.2301 (3)	0.8971 (2)	1.1827 (2)	0.0239 (5)	
C22	0.1156 (3)	0.9462 (3)	1.2565 (2)	0.0334 (6)	
H22	0.0533	1.0248	1.2213	0.040*	
C23	0.0956 (3)	0.8769 (3)	1.3826 (2)	0.0374 (6)	
H23	0.0199	0.9115	1.4309	0.045*	
C24	0.2886 (3)	0.7152 (2)	1.3680 (2)	0.0331 (6)	
H24	0.3473	0.6355	1.4060	0.040*	
C25	0.3188 (3)	0.7786 (2)	1.2413 (2)	0.0283 (5)	
H25	0.3975	0.7426	1.1955	0.034*	
Cl1	0.60963 (8)	0.63418 (6)	0.52615 (5)	0.03639 (17)	
N1	0.6063 (2)	0.78037 (18)	0.69741 (18)	0.0272 (4)	
N2	0.1514 (2)	1.07932 (18)	1.00421 (17)	0.0252 (4)	
N3	−0.2072 (2)	1.47156 (17)	0.77436 (17)	0.0234 (4)	
N4	0.1792 (3)	0.7629 (2)	1.43877 (18)	0.0352 (5)	
O1	0.4167 (2)	0.36497 (18)	0.9094 (2)	0.0572 (6)	
O2	0.3980 (2)	0.57395 (16)	0.83257 (16)	0.0351 (4)	
O3	−0.1565 (3)	0.37407 (17)	1.32170 (15)	0.0459 (5)	
H3A	−0.1605	0.3216	1.3939	0.069*	
O4	−0.0721 (4)	0.20509 (19)	1.2873 (2)	0.0792 (9)	
Zn1	0.59827 (3)	0.60234 (2)	0.71763 (2)	0.02356 (10)	

### Atomic displacement parameters (Å^2^)

**Table:**

	*U*^11^	*U*^22^	*U*^33^	*U*^12^	*U*^13^	*U*^23^
C1	0.0224 (12)	0.0315 (14)	0.0246 (12)	−0.0014 (11)	0.0023 (10)	−0.0128 (11)
C2	0.0208 (11)	0.0233 (12)	0.0210 (11)	−0.0041 (9)	0.0016 (9)	−0.0117 (10)
C3	0.0225 (12)	0.0239 (12)	0.0212 (11)	−0.0017 (9)	−0.0032 (9)	−0.0078 (10)
C4	0.0236 (12)	0.0216 (12)	0.0177 (11)	−0.0052 (9)	0.0018 (9)	−0.0085 (9)
C5	0.0269 (13)	0.0289 (14)	0.0248 (12)	−0.0014 (10)	0.0015 (10)	−0.0074 (11)
C6	0.0296 (13)	0.0293 (13)	0.0285 (12)	−0.0070 (10)	0.0075 (10)	−0.0167 (11)
C7	0.0355 (14)	0.0297 (14)	0.0254 (12)	−0.0126 (11)	0.0064 (10)	−0.0110 (11)
C8	0.0319 (13)	0.0198 (12)	0.0285 (12)	−0.0032 (10)	−0.0028 (10)	−0.0094 (10)
C9	0.0232 (12)	0.0207 (12)	0.0222 (11)	0.0001 (9)	−0.0041 (9)	−0.0092 (10)
C10	0.0277 (13)	0.0223 (12)	0.0221 (11)	−0.0024 (10)	0.0034 (10)	−0.0077 (10)
C11	0.0247 (12)	0.0220 (12)	0.0228 (11)	−0.0017 (9)	−0.0027 (9)	−0.0109 (10)
C12	0.0284 (13)	0.0235 (12)	0.0185 (11)	0.0001 (10)	−0.0017 (9)	−0.0065 (10)
C13	0.0244 (12)	0.0206 (12)	0.0217 (11)	0.0007 (9)	−0.0044 (9)	−0.0078 (9)
C14	0.0242 (12)	0.0199 (12)	0.0228 (11)	0.0002 (9)	−0.0046 (9)	−0.0093 (10)
C15	0.0259 (12)	0.0197 (12)	0.0243 (12)	0.0030 (9)	−0.0051 (10)	−0.0075 (10)
C16	0.0240 (12)	0.0212 (12)	0.0244 (11)	−0.0009 (9)	−0.0039 (9)	−0.0102 (10)
C17	0.0240 (12)	0.0294 (13)	0.0234 (12)	0.0045 (10)	0.0034 (10)	−0.0085 (10)
C18	0.0290 (13)	0.0254 (13)	0.0229 (12)	0.0027 (10)	−0.0004 (10)	−0.0063 (10)
C19	0.0228 (12)	0.0279 (13)	0.0235 (11)	−0.0026 (10)	0.0026 (9)	−0.0145 (10)
C20	0.0301 (13)	0.0227 (12)	0.0207 (11)	−0.0015 (10)	−0.0042 (10)	−0.0088 (10)
C21	0.0255 (12)	0.0231 (12)	0.0216 (11)	−0.0030 (9)	−0.0043 (9)	−0.0089 (10)
C22	0.0314 (14)	0.0353 (15)	0.0274 (13)	0.0053 (11)	−0.0047 (11)	−0.0142 (11)
C23	0.0309 (14)	0.0513 (17)	0.0277 (13)	−0.0027 (12)	0.0020 (11)	−0.0207 (13)
C24	0.0459 (16)	0.0254 (13)	0.0254 (12)	−0.0056 (11)	−0.0115 (11)	−0.0062 (11)
C25	0.0338 (13)	0.0264 (13)	0.0236 (12)	0.0003 (10)	−0.0055 (10)	−0.0122 (10)
Cl1	0.0431 (4)	0.0347 (4)	0.0254 (3)	0.0005 (3)	−0.0060 (3)	−0.0116 (3)
N1	0.0283 (11)	0.0197 (10)	0.0263 (10)	−0.0008 (8)	0.0023 (8)	−0.0094 (8)
N2	0.0246 (10)	0.0228 (10)	0.0229 (10)	0.0026 (8)	−0.0027 (8)	−0.0096 (8)
N3	0.0216 (10)	0.0211 (10)	0.0236 (10)	0.0011 (8)	−0.0020 (8)	−0.0098 (8)
N4	0.0430 (13)	0.0395 (13)	0.0204 (10)	−0.0138 (11)	−0.0020 (9)	−0.0092 (10)
O1	0.0329 (11)	0.0332 (11)	0.0777 (15)	0.0026 (9)	0.0135 (10)	−0.0184 (11)
O2	0.0243 (9)	0.0326 (10)	0.0384 (10)	−0.0087 (8)	0.0103 (8)	−0.0140 (8)
O3	0.0776 (15)	0.0351 (11)	0.0173 (8)	−0.0151 (10)	0.0021 (9)	−0.0076 (8)
O4	0.125 (2)	0.0231 (12)	0.0391 (12)	0.0033 (12)	0.0276 (13)	−0.0023 (9)
Zn1	0.02041 (16)	0.01912 (16)	0.02327 (15)	0.00026 (10)	0.00197 (11)	−0.00766 (11)

### Geometric parameters (Å, º)

**Table:**

C1—O1	1.217 (3)	C14—C15	1.389 (3)
C1—O2	1.277 (3)	C14—C21	1.493 (3)
C1—C2	1.507 (3)	C15—H15	0.9300
C2—C3	1.389 (3)	C16—C20	1.390 (3)
C2—C4^i^	1.399 (3)	C16—C17	1.389 (3)
C3—C4	1.393 (3)	C17—C18	1.366 (3)
C3—H3	0.9300	C17—H17	0.9300
C4—C2^i^	1.399 (3)	C18—N3	1.344 (3)
C4—C5	1.503 (3)	C18—H18	0.9300
C5—O4	1.192 (3)	C19—N3	1.345 (3)
C5—O3	1.298 (3)	C19—C20	1.368 (3)
C6—N1	1.339 (3)	C19—H19	0.9300
C6—C7	1.372 (3)	C20—H20	0.9300
C6—H6	0.9300	C21—C22	1.390 (3)
C7—C8	1.379 (3)	C21—C25	1.392 (3)
C7—H7	0.9300	C22—C23	1.379 (3)
C8—C9	1.390 (3)	C22—H22	0.9300
C8—H8	0.9300	C23—N4	1.333 (3)
C9—C10	1.389 (3)	C23—H23	0.9300
C9—C11	1.488 (3)	C24—N4	1.336 (3)
C10—N1	1.341 (3)	C24—C25	1.379 (3)
C10—H10	0.9300	C24—H24	0.9300
C11—C12	1.390 (3)	C25—H25	0.9300
C11—C15	1.392 (3)	Zn1—Cl1	2.2278 (7)
C12—C13	1.393 (3)	Zn1—N1	2.070 (2)
C12—H12	0.9300	Zn1—O2	1.9171 (16)
C13—N2	1.336 (3)	Zn1—N3^ii^	2.0217 (18)
C13—C16	1.490 (3)	O3—H3A	0.8200
C14—N2	1.343 (3)		
			
O1—C1—O2	125.8 (2)	C20—C16—C13	120.1 (2)
O1—C1—C2	120.4 (2)	C17—C16—C13	122.8 (2)
O2—C1—C2	113.6 (2)	C18—C17—C16	119.9 (2)
C3—C2—C4^i^	119.3 (2)	C18—C17—H17	120.1
C3—C2—C1	118.5 (2)	C16—C17—H17	120.1
C4^i^—C2—C1	122.0 (2)	N3—C18—C17	123.1 (2)
C2—C3—C4	121.4 (2)	N3—C18—H18	118.4
C2—C3—H3	119.3	C17—C18—H18	118.4
C4—C3—H3	119.3	N3—C19—C20	123.1 (2)
C3—C4—C2^i^	119.3 (2)	N3—C19—H19	118.5
C3—C4—C5	117.6 (2)	C20—C19—H19	118.5
C2^i^—C4—C5	122.9 (2)	C19—C20—C16	119.8 (2)
O4—C5—O3	124.4 (2)	C19—C20—H20	120.1
O4—C5—C4	124.0 (2)	C16—C20—H20	120.1
O3—C5—C4	111.4 (2)	C22—C21—C25	117.5 (2)
N1—C6—C7	122.9 (2)	C22—C21—C14	120.1 (2)
N1—C6—H6	118.5	C25—C21—C14	122.4 (2)
C7—C6—H6	118.5	C23—C22—C21	119.2 (2)
C6—C7—C8	118.5 (2)	C23—C22—H22	120.4
C6—C7—H7	120.7	C21—C22—H22	120.4
C8—C7—H7	120.7	N4—C23—C22	123.4 (2)
C7—C8—C9	120.0 (2)	N4—C23—H23	118.3
C7—C8—H8	120.0	C22—C23—H23	118.3
C9—C8—H8	120.0	N4—C24—C25	123.1 (2)
C8—C9—C10	117.3 (2)	N4—C24—H24	118.4
C8—C9—C11	121.7 (2)	C25—C24—H24	118.4
C10—C9—C11	121.0 (2)	C24—C25—C21	119.3 (2)
N1—C10—C9	123.1 (2)	C24—C25—H25	120.4
N1—C10—H10	118.5	C21—C25—H25	120.4
C9—C10—H10	118.5	C6—N1—C10	118.1 (2)
C12—C11—C15	118.0 (2)	C6—N1—Zn1	119.29 (16)
C12—C11—C9	120.2 (2)	C10—N1—Zn1	121.30 (16)
C15—C11—C9	121.8 (2)	C13—N2—C14	118.72 (19)
C11—C12—C13	119.4 (2)	C18—N3—C19	116.97 (19)
C11—C12—H12	120.3	C18—N3—Zn1^iii^	120.55 (15)
C13—C12—H12	120.3	C19—N3—Zn1^iii^	121.90 (15)
N2—C13—C12	122.3 (2)	C23—N4—C24	117.5 (2)
N2—C13—C16	115.34 (19)	C1—O2—Zn1	125.37 (16)
C12—C13—C16	122.25 (19)	C5—O3—H3A	109.5
N2—C14—C15	122.2 (2)	O2—Zn1—N3^ii^	115.73 (8)
N2—C14—C21	114.85 (19)	O2—Zn1—N1	97.21 (8)
C15—C14—C21	122.9 (2)	N3^ii^—Zn1—N1	115.16 (8)
C11—C15—C14	119.4 (2)	O2—Zn1—Cl1	119.86 (6)
C11—C15—H15	120.3	N3^ii^—Zn1—Cl1	104.98 (6)
C14—C15—H15	120.3	N1—Zn1—Cl1	103.51 (6)
C20—C16—C17	117.0 (2)		
			
O1—C1—C2—C3	38.5 (3)	C17—C16—C20—C19	3.6 (3)
O2—C1—C2—C3	−137.7 (2)	C13—C16—C20—C19	−174.3 (2)
O1—C1—C2—C4^i^	−147.7 (3)	N2—C14—C21—C22	−1.5 (3)
O2—C1—C2—C4^i^	36.1 (3)	C15—C14—C21—C22	180.0 (2)
C4^i^—C2—C3—C4	−1.3 (4)	N2—C14—C21—C25	177.0 (2)
C1—C2—C3—C4	172.7 (2)	C15—C14—C21—C25	−1.6 (4)
C2—C3—C4—C2^i^	1.3 (4)	C25—C21—C22—C23	0.3 (4)
C2—C3—C4—C5	−173.6 (2)	C14—C21—C22—C23	178.9 (2)
C3—C4—C5—O4	−53.0 (4)	C21—C22—C23—N4	−1.1 (4)
C2^i^—C4—C5—O4	132.3 (3)	N4—C24—C25—C21	−1.2 (4)
C3—C4—C5—O3	123.2 (2)	C22—C21—C25—C24	0.7 (4)
C2^i^—C4—C5—O3	−51.5 (3)	C14—C21—C25—C24	−177.8 (2)
N1—C6—C7—C8	−0.1 (4)	C7—C6—N1—C10	−1.8 (4)
C6—C7—C8—C9	2.3 (4)	C7—C6—N1—Zn1	165.3 (2)
C7—C8—C9—C10	−2.5 (3)	C9—C10—N1—C6	1.6 (4)
C7—C8—C9—C11	−179.9 (2)	C9—C10—N1—Zn1	−165.17 (18)
C8—C9—C10—N1	0.5 (4)	C12—C13—N2—C14	1.0 (4)
C11—C9—C10—N1	177.9 (2)	C16—C13—N2—C14	176.5 (2)
C8—C9—C11—C12	38.0 (3)	C15—C14—N2—C13	−0.4 (3)
C10—C9—C11—C12	−139.3 (2)	C21—C14—N2—C13	−178.9 (2)
C8—C9—C11—C15	−143.1 (2)	C17—C18—N3—C19	2.2 (4)
C10—C9—C11—C15	39.5 (3)	C17—C18—N3—Zn1^iii^	−169.2 (2)
C15—C11—C12—C13	−1.1 (3)	C20—C19—N3—C18	−2.1 (3)
C9—C11—C12—C13	177.8 (2)	C20—C19—N3—Zn1^iii^	169.18 (18)
C11—C12—C13—N2	−0.3 (4)	C22—C23—N4—C24	0.7 (4)
C11—C12—C13—C16	−175.5 (2)	C25—C24—N4—C23	0.4 (4)
C12—C11—C15—C14	1.7 (3)	O1—C1—O2—Zn1	22.1 (4)
C9—C11—C15—C14	−177.2 (2)	C2—C1—O2—Zn1	−161.85 (15)
N2—C14—C15—C11	−1.0 (4)	C1—O2—Zn1—N3^ii^	−49.0 (2)
C21—C14—C15—C11	177.4 (2)	C1—O2—Zn1—N1	−171.5 (2)
N2—C13—C16—C20	−11.3 (3)	C1—O2—Zn1—Cl1	78.4 (2)
C12—C13—C16—C20	164.2 (2)	C6—N1—Zn1—O2	−152.28 (19)
N2—C13—C16—C17	170.8 (2)	C10—N1—Zn1—O2	14.38 (19)
C12—C13—C16—C17	−13.7 (4)	C6—N1—Zn1—N3^ii^	84.84 (19)
C20—C16—C17—C18	−3.6 (4)	C10—N1—Zn1—N3^ii^	−108.50 (18)
C13—C16—C17—C18	174.4 (2)	C6—N1—Zn1—Cl1	−29.16 (19)
C16—C17—C18—N3	0.7 (4)	C10—N1—Zn1—Cl1	137.50 (17)
N3—C19—C20—C16	−0.8 (4)		

Symmetry codes: (i) −*x*, −*y*+1, −*z*+2; (ii) *x*+1, *y*−1, *z*; (iii) *x*−1, *y*+1, *z*.

### Hydrogen-bond geometry (Å, º)

**Table:**

*D*—H···*A*	*D*—H	H···*A*	*D*···*A*	*D*—H···*A*
O3—H3*A*···N4^iv^	0.82	1.83	2.633 (3)	167
C15—H15···O1^v^	0.93	2.46	3.383 (3)	172
C17—H17···Cl1^vi^	0.93	2.75	3.678 (3)	173
C22—H22···O4^vii^	0.93	2.59	3.305 (4)	134
C25—H25···O1^v^	0.93	2.34	3.267 (3)	175

Symmetry codes: (iv) −*x*, −*y*+1, −*z*+3; (v) −*x*+1, −*y*+1, −*z*+2; (vi) −*x*+1, −*y*+2, −*z*+1; (vii) *x*, *y*+1, *z*.

## References

[bb1] Bhaumik, C., Saha, D., Das, S. & Baitalik, S. (2011). *Inorg. Chem.* **50**, 12586–12600. 10.1021/ic201610w 22098484

[bb2] Brammer, L. (2004). *Chem. Soc. Rev.* **33**, 476–489.

[bb3] Bruker (2000). *SMART*, *SAINT* and *SADABS*. Bruker AXS Inc., Madison, Wisconsin, USA.

[bb4] Dai, F., He, H. & Sun, D. (2008). *J. Am. Chem. Soc.* **130**, 14064–14065. 10.1021/ja805920t 18831586

[bb5] Hancock, R. D. (2013). *Chem. Soc. Rev.* **42**, 1500–1524.

[bb6] Hinterholzinger, F. M., Ranft, A., Feckl, J. M., Rühle, B., Bein, T. & Lotsch, B. V. (2012). *J. Mater. Chem.* **22**, 10356–10362.

[bb7] Hou, K.-L., Bai, F.-Y., Xing, Y.-H., Wang, J.-L. & Shi, Z. (2011). *CrystEngComm*, **13**, 3884–3894.

[bb8] Housecroft, C. E. (2014). *Dalton Trans.* **43**, 6594–6604. 10.1039/c4dt00074a 24522847

[bb9] Jiang, H.-L., Makal, T. A. & Zhou, H.-C. (2013). *Coord. Chem. Rev.* **257**, 2232–2249.

[bb10] Kepert, C. J. (2006). *Chem. Commun.* pp. 695–700. 10.1039/b515713g 16465312

[bb11] Kreno, L. E., Leong, K., Farha, O. K., Allendorf, M., Van Duyne, R. P. & Hupp, J. T. (2012). *Chem. Rev.* **112**, 1105–1125. 10.1021/cr200324t 22070233

[bb12] Kumazawa, K., Biradha, K., Kusukawa, T., Okano, T. & Fujita, M. (2003). *Angew. Chem. Int. Ed.* **42**, 3909–3913. 10.1002/anie.200351797 12949866

[bb13] Li, M.-X., Miao, Z.-X., Shao, M., Liang, S.-W. & Zhu, S.-R. (2008). *Inorg. Chem.* **47**, 4481–4489. 10.1021/ic701346x 18459771

[bb14] Li, J.-R., Sculley, J. & Zhou, H.-C. (2012). *Chem. Rev.* **112**, 869–932. 10.1021/cr200190s 21978134

[bb15] Li, X.-Z., Zhou, X.-P., Li, D. & Yin, Y.-G. (2011). *CrystEngComm*, **13**, 6759–6765.

[bb16] Ma, B.-Q. & Coppens, P. (2003). *Chem. Commun.* pp. 2290–2291. 10.1039/b306965f 14518879

[bb17] Rosi, N. L., Eckert, J., Eddaoudi, M., Vodak, D. T., Kim, J., O’Keeffe, M. & Yaghi, O. M. (2003). *Science*, **300**, 1127–1129. 10.1126/science.1083440 12750515

[bb18] Seo, J. S., Whang, D., Lee, H., Jun, S. I., Oh, J., Jeon, Y. J. & Kim, K. (2000). *Nature*, **404**, 982–986. 10.1038/35010088 10801124

[bb19] Sheldrick, G. M. (2008). *Acta Cryst.* A**64**, 112–122. 10.1107/S0108767307043930 18156677

[bb20] Sheldrick, G. M. (2015). *Acta Cryst.* C**71**, 3–8.

[bb21] Wang, B.-C., Chen, X.-L., Hu, H.-M., Yao, H.-L. & Xue, G.-L. (2009). *Inorg. Chem. Commun.* **12**, 856–859.

[bb22] Yaghi, O. M. & Li, H. (1995). *J. Am. Chem. Soc.* **117**, 10401–10402.

[bb23] Yang, X.-L., Shangguan, Y.-Q., Hu, H.-M., Xu, B., Wang, B.-C., Xie, J., Yuan, F., Yang, M.-L., Dong, F.-X. & Xue, G.-L. (2014). *J. Solid State Chem.* **216**, 13–22.

[bb24] Yoshizawa, M., Miyagi, S., Kawano, M., Ishiguro, K. & Fujita, M. (2004). *J. Am. Chem. Soc.* **126**, 9172–9173. 10.1021/ja047612u 15281793

